# 

**Published:** 1909-03-06

**Authors:** 


### GRADUATES' MEDICAL SCHOOLS.

DIARY FOR THE WEEK MARCH 8 TO MARCH 13.

**NORTH-EAST LONDON POST-GRADUATE COLLEGE, Prince of Wales's General Hospital, Tottenham, N.**

**At 4.30 p.m.**

**March 9,** Dr. Murray Leslie, **Modern Views on Heredity.**

**March II,** Dr. G. G. Macdonald, **The Evolution of Disease.**

**THE HOSPITAL FOR SICK CHILDREN, Great Ormond Street, W.C.**

**At 4 p.m.**

**March II,** Dr. Poynton, **Amaurotic Family Idiocy.**

**MEDICAL GRADUATES' COLLEGE AND POLYCLINIC, 22 Chenies Street, W.C.**

**At 5.15 p.m.**

**March 8,** Mr. E. Clarke, **The Fundus Oculi.**

**March 9,** Dr. F. J. McCann, **Pruritus Vulvæ: Its Causes and Treatment.**

**March IO,** Mr. R. H. J. Swan, **Vesical Tuberculosis.**

**March II,** Dr. J. M. H. MacLeod, **Bullous Eruptions.**

**THE THROAT HOSPITAL, Golden Square, W.**

**At 5.30 p.m**

**March 8,** Mr. Parker, **Neuroses of the Larynx.**

**March II,** Dr. Powell, **New Growths of the Larynx.**

**NATIONAL HOSPITAL FOR THE PARALYSED AND EPILEPTIC, Queen Sq., Bloomsbury, W.C.**

**At 3.30 p.m.**

**March 9.** Dr. Tooth, **Clinical Lecture.**

**March 12,** Mr. Armour, **Symptoms and Treatment of Cervical Ribs.**

**LONDON SCHOOL OF CLINICAL MEDICINE, Seamen's Hospital, Greenwich, S.E.**

**At 3.15 p.m.**

**March 8,** Mr. Turner, **Difficulties of Abdominal Diagnosis.**

**At 2.15 p.m.**

**March 9,** Dr. Russell Wells, **Mitral Disease.**

**At 3.30 p.m.**

**March IO,** Mr. Cargill, **Papillitis.**

**THE POST-GRADUATE COLLEGE, West London Hospital, Hammersmith, W.**

**At IO a.m.**

**March 8 and II,** Surgical Registrar, **Demonstration.**

**March 12,** Medical Registrar, **Demonstration.**

**At I2 noon.**

**March 8,** Dr. Low, **Pathological Demonstration.**

**At 12.15 p.m.**

**March IO and I3,** Dr. Pritchard, **Practical Medicine.**

**At 5 p.m.**

**March 8,** Dr. Pritchard, **Clinical Pathology.**

**March 9,** Mr. Bidwell, **Ulceration of the Cæcum.**

**March IO,** Dr. Beddard, **Medicine—IV.**

**March II,** Mr. Lloyd Williams, **Swellings of the Jaws.**

**March I2,** Dr. Seymour Taylor, **Treatment of Constipation.**

**LONDON THROAT HOSPITAL, Great Portland Street, W.**

**At 5 p.m.**

**March IO,** Dr. Kelson, **Surgical Landmarks of the Nose and Throat.**

**ST. JOHN'S HOSPITAL FOR DISEASES OF THE SKIN, Leicester Square, W.C.**

**March II,** Chesterfield Lectures, **Ul-erythema.**

THE HOSPITAL March 6, 1909.

*Name* ..........................................................................................

*Address* ......................................................................................

..........................................................................................

This Coupon must accompany manuscript or contributions intended for The Hospital.

